# Mental Health Symptoms Among Chinese College Students Following the Lifting of COVID-19 Restrictions: A Serial Cross-Sectional Study in Guangdong Province, China

**DOI:** 10.3390/healthcare14030339

**Published:** 2026-01-29

**Authors:** Zijie Ma, Yujing Chen, Yishuai Deng, Jingbo Zhao

**Affiliations:** 1 Mental Health Education and Counseling Center, School of Public Health, Southern Medical University, Guangzhou 510515, China; 2 Department of Psychology, School of Public Health, Southern Medical University, Guangzhou 510515, China

**Keywords:** mental health, students, COVID-19, depression, anxiety, cross-sectional studies

## Abstract

Background/Objectives: To assess the mental health of Chinese college students following the lifting of COVID-19 restrictions in December 2022. Methods: A three-wave cross-sectional study was conducted among students from 22 colleges in Guangdong, China, at three time points: Onset of COVID-19 (February 2020; T1; initial survey wave), during restrictions (June 2021; T2), and after restrictions (March to April 2023; T3). Participants at each wave were 164,101, 86,767, and 130,285, respectively. The standardized prevalence rates of depression, suicidal ideation (SI), anxiety, insomnia, acute stress, and fear of COVID-19 after restrictions were compared with those from the initial survey wave and restriction periods. Multivariate logistic regression was used to identify associated risk factors. Results: After restrictions were lifted, standardized prevalence rates of mental symptoms were as follows: anxiety (13.5%), depression (19.9%), insomnia (11.8%), acute stress (19.7%), fear of COVID-19 (16.2%), and suicidal ideation (31.8%). The standardized prevalence rates at T3 were higher than those at T1, with absolute increases of 10.9% for anxiety, 13.9% for depression, 9.1% for insomnia, and 23.5% for suicidal ideation. Acute stress showed a V-shaped pattern, with lower prevalence during the restriction period compared to T1, followed by an increase at T3. Fear of COVID-19 declined after the initial phase and remained stable. Students with a history of infection, those perceiving greater pandemic impact, and those who either neglected or excessively engaged in protective behaviors were at elevated risk for mental health symptoms. Conclusions: Our findings highlight the long-term adverse effects of the pandemic at the population level on Chinese college students’ mental health. Continuous monitoring, early prevention, and accessible mental health care should be prioritized in the coming years.

## 1. Introduction

There is growing evidence of the long-term and serious impact of the COVID-19 pandemic on mental health, including its potential to trigger lasting psychological disorders [1,2]. College students, due to their heightened vulnerability to psychological stress, have been identified as a high-risk group during the pandemic [3,4].

Chinese students have generally experienced three distinct phases of the COVID-19 pandemic: (1) Onset of the COVID-19 pandemic. Beginning in late 2019, the pandemic rapidly spread worldwide, profoundly altering daily life [5]. In the early months, studies already reported high prevalence rates of anxiety, depression, acute stress, fear, and suicidal ideation (SI) among students [2,6,7,8,9]. (2) During restrictions. During this phase, strict control measures were normalized. Students were often quarantined on campus and subjected to lockdowns that disrupted their usual social interactions [10]. Longitudinal research indicated rising prevalence rates of mental health symptoms during this time [11,12], except for acute stress and fear, which reportedly declined [2,8], which was found to decrease. (3) After restrictions. In December 2022, China eased its COVID-19 policies to mitigate the prolonged effects of mass lockdowns [13]. New guidelines significantly relaxed mobility restrictions, requiring schools without outbreaks to resume in-person teaching [14]. Following these changes, many students were infected, further disrupting their social lives. Some experienced the loss of friends or family members due to the virus, compounding their psychological distress. A recent study found that depression, anxiety, insomnia, and PTSD were common among college students after restrictions were lifted [15]. In light of this large-scale infection, it remains essential to investigate students’ mental responses and compare their psychological well-being across different stages of the pandemic.

The COVID-19 pandemic has been characterized by multiple waves of outbreaks and shifting public health strategies. While many studies have examined the pandemic’s impact on students’ mental health, systematic assessments focusing on the distinct psychosocial context of the after-restriction phase—a period marked by widespread personal infection, societal reopening, and resumption of academic pressures [4]—remain scarce. Furthermore, few studies have directly compared the psychological burden of this phase with those of the initial outbreak and prolonged restriction periods using standardized metrics, which is crucial for understanding the evolving, phase-specific impact of the pandemic. To address these gaps, we conducted a large-scale serial cross-sectional survey spanning all three critical phases (onset, during restrictions, and after restrictions). Our study has two primary objectives: First, to assess the prevalence and identify the unique risk factors (e.g., infection status, post-infection health concerns) associated with common mental health symptoms among Chinese college students, specifically after the lifting of COVID-19 restrictions. Second, and more importantly, to use gender- and degree-standardized prevalence rates to systematically compare the mental health burden of this after-restriction phase with the two earlier stages. This approach moves beyond tracking prevalence within a single phase; it aims to delineate how the population-level psychological burden of the pandemic transformed alongside major policy and societal shifts. By employing standardized rates for cross-phase comparison, our study provides a novel, population-level lens through which to assess the distinct mental health impact of the after-restriction transition. The findings aim to offer timely evidence to guide targeted mental health support and policy planning for students in the post-pandemic era.

## 2. Materials and Methods

### 2.1. Study Design and Participants

This study employed a serial cross-sectional design to assess mental health trends at three distinct phases of the COVID-19 pandemic. A convenience sampling method was used, given the large-scale, multi-institutional nature of the survey. Online questionnaires were distributed to 22 universities using the Sojump platform (an online survey tool) via web links or QR codes. All respondents provided electronic informed consent on the first page of the questionnaire. They were explicitly informed of their right to withdraw at any time without penalty.

**Figure 1:**
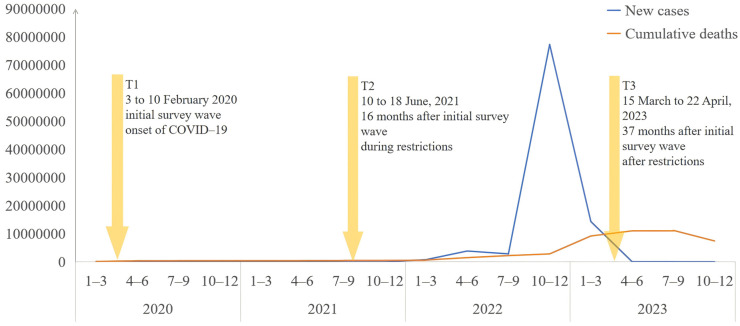
Trend of new COVID-19 cases and cumulative deaths in China from January 2020 to December 2023. Note: Data retrieved from https://data.who.int/dashboards/covid19/cases?n=c (accessed on 24 February 2024).

To identify and exclude ineligible participants, two screening criteria were applied: (1) total response time less than 289 s (our questionnaire contained 5280 characters, and the maximum reading speed for Chinese characters is 1097 characters per minute), and (2) uniform responses to all questions throughout the survey. Data was collected at three time points: from 3 to 10 February 2020 (T1, initial survey wave, onset of COVID-19), 10 to 18 June 2021 (T2, 16 months after initial survey wave, during restrictions), 15 March to 22 April 2023 (T3, 37 months after initial survey wave, after restrictions). Figure 1 illustrates the trend of COVID-19 cases in China from January 2020 to December 2023. Across the three waves, we distributed 185,845 (T1), 93,413 (T2), and 148,897 (T3) questionnaires, yielding 164,101, 86,767, and 130,285 valid responses, respectively. The corresponding effective response rates were 88.3%, 92.8%, and 87.5%. The total analytical sample across all waves consisted of 381,152 participants. Details of the survey time points are as follows:

(1) T1 (3–10 February 2020): This period marked the initial survey wave of the initial COVID-19 outbreak in China, prior to the implementation of standardized prevention measures. By 2 February, there were 2829 confirmed cases and 57 deaths. All 31 mainland provinces activated Level 1 emergency responses. The government imposed strict controls, including home quarantine for close contacts, cancelation of public events, and a delay in college students’ return to campus. (2) T2 (10–18 June 2021): Sixteen months after the initial survey wave, this stage focused on achieving zero new cases. During the survey, a Delta variant outbreak was reported in Guangzhou on 21 May. One university reported infections and shifted to online learning and home isolation. Other universities without confirmed cases continued in-person classes. Thus, colleges implemented hierarchical management and adapted prevention strategies based on their individual situations. (3) T3 (15 March–22 April 2023): Thirty-seven months after the initial survey wave, this stage followed China’s policy adjustments regarding COVID-19. Routine testing and quarantine measures were relaxed, and the government promoted self-health management while resuming regular activities and large-scale gatherings. Our survey coincided with students’ first return to campus after-restrictions, during which many had experienced COVID-19 infection.

This study was approved by the Human Research Ethics Committee of South China Normal University (Ethics No. SCNU-PSY-2020-01-001). All procedures adhered to the principles of the Declaration of Helsinki.

### 2.2. Methods

#### 2.2.1. Demographics

The demographic information collected included: (1) age, (2) sex (male or female), (3) living area (urban, rural), (4) grade (undergraduate, postgraduate), (5) severe somatic disease (yes or no), (6) mental disorder (yes or no), (7) history of psychotropic drug use (yes or no). We also collected some information about COVID-19: (1) infection (uninfected, never been infected from T1 to T3; under infection, been infected during T3; infected, been infected during T1 or T2 while without the perception of recovery, or having sequelae during T3; recovered from infection, sense of recovery from the infection at T3), (2) whether they were concerned more about their health after COVID-19 infection (yes or no), (3) time spent on surfing information about COVID-19 (≤1 h, 1–3 h, 3–5 h, ≥5 h), (4) perceived impacts on their family incomes, and (5) personal development, such as academic or career development (e.g., Has COVID-19 affected your family incomes/personal development: lesser impact, large impact, severe impact), (6) COVID-19 prevention behaviors (without prevention, moderate prevention, much prevention).

#### 2.2.2. Mental Health Symptoms

The following symptoms were assessed: (1) Anxiety, measured using the 7-item Generalized Anxiety Disorder Scale (GAD-7), which evaluates anxiety symptoms over the past two weeks. Each item is rated on a 4-point Likert scale from 0 (“not at all”) to 3 (“nearly every day”). Total scores range from 0 to 21, with higher scores indicating greater anxiety severity [16]. In the present sample, Cronbach’s α was 0.92. (2) Depression, measured using the 8-item Patient Health Questionnaire (PHQ-8), to assess depressive symptoms over the past two weeks. Items are scored from 0 (“not at all”) to 3 (“nearly every day”). Total scores range from 0 to 24 [17]. In this study, Cronbach’s α was 0.89. (3) Insomnia, assessed using the 8-item Youth Self-Rating Insomnia Scale (YSIS), which measures the severity of current insomnia symptoms. Responses are rated on a 5-point scale, with total scores ranging from 8 to 40 [18]. In our sample, Cronbach’s α was 0.86. (4) Acute stress, measured using the 6-item Impact of Event Scale (IES-6), which evaluates acute stress reactions during the past 7 days. Responses are rated on a 4-point Likert scale ranging from 0 to 3 (0 = not at all; 1 = several days; 2 = more than half the days; 3 = nearly every day). The summed score ranges from 0 to 18, with higher scores indicating greater severity of acute stress [19,20]. Cronbach’s α in this study was 0.82. (5) Fear of COVID-19, assessed using the 12-item COVID-19 Fear Screening Scale (CV-19FSS), adapted from the SARS Fear Emotion Screening Inventory to evaluate fear related to COVID-19. Items are scored dichotomously (0 = no, 1 = yes) [8,21]. In our sample, Cronbach’s α was 0.80. (6) Suicidal ideation (SI), assessed using the 9th item of the 9-item Patient Health Questionnaire (PHQ-9). The item is scored from 0 (“not at all”) to 3 (“nearly every day”). Any response other than 0 (i.e., a score ≥ 1) was used to indicate the presence of suicidal ideation [22]. Previous research supports the 9th item of the PHQ-9 as a valid screening tool for SI [23,24].

The clinical thresholds used to define each symptom (GAD-7 ≥ 10, PHQ-8 ≥ 10, YSIS ≥ 26, IES-6 ≥ 9, CV-19FSS ≥ 4, SI ≥ 1) were based on established cut-offs validated in previous studies [8,16,17,18,19,20,21,22,23,24], thereby ensuring the validity and comparability of our case definitions. All instruments, cut-off scores, and administration procedures were held strictly consistent across all three survey waves (T1, T2, and T3) to ensure the comparability of prevalence estimates over time.

### 2.3. Statistical Analyses

First, as most measurement tools and quantitative covariates were not normally distributed, we described the sample using medians with interquartile ranges (IQRs). To enhance comparability of prevalence estimates across the three independent cross-sectional samples (T1, T2, T3), we calculated gender- and degree-standardized prevalence rates. Standardization was performed using the direct method, based on the demographic structure (gender and degree distribution) of the college student population in Guangdong Province for the academic years 2020–2021, as published by the Ministry of Education of the People’s Republic of China [25]. This involved calculating the prevalence that would have been observed in each wave if its sample had the same proportions of males/females and undergraduates/postgraduates as the provincial reference population. This approach adjusts for potential demographic differences between waves and allows for more meaningful comparison of population-level trends over time [4]. Using these standardized rates, we compared the prevalence of mental health symptoms at T3 with those at T1 and T2. During the calculation of standardized prevalence rates, 13 participants were excluded at T3 due to missing grade information. After exclusion, the standardized prevalence rates and corresponding confidence intervals remained unchanged. Chi-square tests were used for pairwise comparisons of standardized prevalence rates (i.e., T1 vs. T2, T2 vs. T3, T1 vs. T3). Bivariate analyses were conducted using chi-square tests to examine associations between mental symptoms and covariates. Multivariate logistic regression models were then used to identify risk factors associated with reporting at least one mental health symptom at T3. Similar models were subsequently applied to each individual outcome. Strength of associations was presented using odds ratios (ORs) with 95% confidence intervals (CIs).

All data analyses were conducted using SPSS version 25.0. Standardized prevalence rates were calculated by R 3.6.0. A significance level of 0.05 was adopted, and all tests were two-sided.

## 3. Results

### 3.1. Demographic Characteristics

At T3, our sample included 76,721 (58.9%) female participants. The median age was 20 years (IQR: 19–21). The majority were undergraduates (123,030; 94.4%). A total of 46,782 (35.9%) participants resided in rural areas. Additionally, 1.3% (1678) reported having severe somatic diseases, 2.0% (2657) had mental disorders, and 4.0% (5169) had a history of psychotropic drug use.

Only 28,357 (21.8%) participants had not been infected by COVID-19. A total of 83,872 (64.4%) reported increased concern about their health following infection. Most participants spent less than 3 h per day searching for COVID-19-related information, with 103,134 (79.2%) spending ≤1 h and 13,266 (10.2%) spending 1–3 h daily. Among all participants, 10,555 (8.1%) perceived a severe impact on their family income, and 10,999 (8.4%) reported a severe impact on their personal development. Additionally, 45,937 (35.3%) reported not taking any protective measures against COVID-19 (See Table 1).

At T3, the sample comprised 76,721 (58.9%) females, with a median age of 20 years (IQR: 19–21); 94.4% (123,030) were undergraduates and 35.9% (46,782) rural residents. Severe somatic diseases, mental disorders and psychotropic drug use history were reported by 1.3% (1678), 2.0% (2657) and 4.0% (5169), respectively.

### 3.2. Prevalence Rates of Mental Health Outcomes

Crude and standardized prevalence rates at T3 are presented in Table 2 (for crude and standardized prevalence rates at T1 and T2, see Tables S1 and S2). At T3, the crude prevalence rates for anxiety, depression, insomnia, acute stress, fear, and suicidal ideation (SI) were 13.8% (95% CI, 13.6–14.0%), 20.4% (95% CI, 20.2–20.6%), 11.8% (95% CI, 11.7–12.0%), 19.8% (95% CI, 19.6–20.0%), 16.9% (95% CI, 16.7–17.1%), and 32.3% (95% CI, 32.0–32.5%), respectively. After adjusting for gender and degree, prevalence rates slightly decreased: (13.5% [95% CI, 13.3–13.7%] for anxiety; 19.9% [95% CI, 19.6–20.1%] for depression; 11.8% [95% CI, 11.6–12.0%] for insomnia; 19.7% [95% CI, 19.5–20.0] for acute stress; 16.2% [95% CI, 16.0–16.4%] for fear; 31.8% [95% CI, 31.5–32.1%] for SI). The medians (IQRs) of the scores were 5 (1–7) for the GAD-7 (anxiety), 5 (1–7) for the PHQ-8 (depression), 19 (16–23) for the YSIS (insomnia), 3 (0–7) for the IES-6 (acute stress), 1 (0–3) for the CV-19FSS (fear), 1 (1–2) for SI.

**Figure 2:**
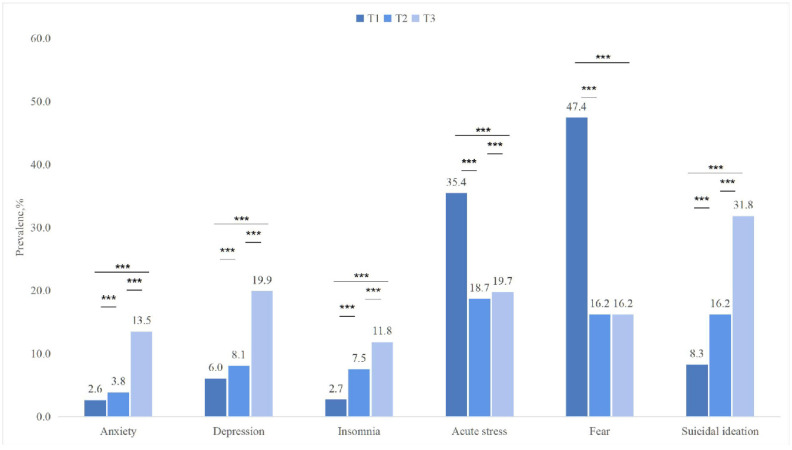
Standardized prevalence rates of mental health disorders during the onset of COVID-19 (T1), during the restrictions (T2), and after the restrictions (T3). Note: *** *p* < 0.001.

Figure 2 presents the standardized prevalence rates measured at T3 along with those measured at T1 and T2. At the population level, the standardized prevalence rates of anxiety, depression, insomnia, and SI were higher at T3 compared to T1 and T2: anxiety rates increased from 2.6% to 13.5%, depression increased from 6.0% to 19.9%, insomnia increased from 2.7% to 11.8%, and SI increased from 8.3% to 31.8%. Standardized prevalence of acute stress showed a V-shaped pattern across the three time points, with a decrease at T2 (18.7%) followed by an increase at T3 (19.7%). The prevalence of fear was highest at T1 (47.4%) and dropped significantly at T2 (16.2%) and remained at that level at T3 (16.2%). All comparisons were statistically significant (*p* < 0.001), except for the comparison between fear prevalence at T2 and T3 (*p* = 0.36).

### 3.3. Factors Associated with Mental Health Outcomes

Bivariate analyses are presented in Table 3, and multivariate analyses in Table 4.

#### 3.3.1. Risk Factors of Demographic Characteristics

Being a postgraduate was associated with lower risks of depression, anxiety, SI, fear, and acute stress (ORs ranged from 0.43 [95% CI, 0.39–0.46] for acute stress to 0.75 [95% CI, 0.69–0.81] for anxiety), but a higher risk of insomnia.

Females had increased risks of anxiety, depression, insomnia, and fear (ORs ranged from 1.13 [95% CI, 1.09–1.16] for fear to 1.19 [95% CI, 1.14–1.23] for insomnia), but decreased risks of SI (adjusted OR, 0.80; 95% CI, 0.78–0.82) and acute stress (adjusted OR, 0.59; 95% CI, 0.57–0.61). Participants living in rural areas reported higher levels of acute stress, fear, and SI (ORs ranged from 1.09 [95% CI, 1.06–1.11] for SI to 1.25 [95% CI, 1.21–1.29] for acute stress).

Students with severe somatic diseases had an increased risk of fear (adjusted OR, 1.77; 95% CI, 1.58–1.99). In addition, those with mental disorders had higher risks for all outcomes (ORs ranged from 1.30 [95% CI, 1.16–1.46] for fear to 1.71 [95% CI, 1.54–1.88]). Participants with a history of psychotropic drug use also showed increased risks (ORs ranged from 1.09 [95% CI, 1.00–1.19] for acute stress to 2.42 [95% CI, 2.24–2.61] for anxiety).

#### 3.3.2. COVID-19-Related Factors

Students who had recovered from COVID-19 had lower risks for all mental health outcomes except insomnia (ORs ranged from 0.83 [95% CI, 0.80–0.87] for fear to 0.95 [95% CI, 0.92–0.99] for anxiety). Participants who were less concerned about their health after infection reported higher levels of anxiety, depression, insomnia, and SI (ORs ranged from 1.28 [95% CI, 1.24–1.33] for insomnia to 1.56 [95% CI, 1.51–1.60] for depression). The more time students spent browsing COVID-19-related information online, the greater the risk of poor mental health outcomes. For example, acute stress among those who spent 3–5 h per day searching for such information had an adjusted OR of 2.42 (95% CI, 2.28–2.57). Students who reported that their family income was severely impacted by COVID-19 were 1.80 to 2.23 times more likely to experience mental health symptoms than those reporting a lesser impact. Similarly, higher odds were found among those who reported that their personal development had been affected (e.g., OR = 2.23 [95% CI, 2.12–2.35] for depression; OR = 2.53 [95% CI, 2.39–2.68] for anxiety).

Students who did not adopt any COVID-19 protective measures were also at higher risk for anxiety, depression, and insomnia (ORs ranged from 1.13 [95% CI, 1.09–1.17] for anxiety to 1.18 [95% CI, 1.14–1.23] for insomnia). Meanwhile, students who reported engaging in excessive protective behaviors showed higher risks for poor mental health outcomes—excluding insomnia—with ORs ranging from 1.05 [95% CI, 1.01–1.10] for depression to 1.95 [95% CI, 1.87–2.03] for fear.

## 4. Discussion

In the current study, we found that after the lifting of COVID-19 restrictions, population-level estimates indicated a worsening of mental health status among college students. By comparing these results with two previous time points, we observed higher prevalence rates at T3 compared to earlier phases for anxiety, depression, insomnia, and suicidal ideation. A V-shaped pattern was noted in acute stress, showing a decline followed by an increase after the onset of COVID-19. Fear of COVID-19 was significantly elevated at the onset of the pandemic, with nearly half of the students reporting concern. This rate declined during the restriction period and remained stable thereafter. Overall, these findings suggest an association between the pandemic phases and mental health symptom prevalence. Additionally, we discussed various associated risk factors.

Comparisons with other studies are complex due to the influence of differences in prevention and control measures, as well as substantial methodological and sample heterogeneity [4,26]. For instance, pre-pandemic estimates for Chinese college students have been reported using various instruments and thresholds: depression prevalence ranged from approximately 7.7% to 29%, anxiety was around 21% among medical students and suicidal ideation was ranged from 9.1% to 10.7% [27,28,29]. While our post-restriction prevalence of depression (19.9%) and anxiety (13.5%) fall within the broad spectrum of these historical estimates, and our suicidal ideation rate (31.8%) appears substantially higher, such numerical comparisons must be interpreted with caution. The primary value of our study, therefore, lies in systematically documenting relative changes across three defined pandemic phases using a consistent methodology within the same population. This internal comparison approach minimizes methodological confounds and more clearly highlights that the psychological burden during the post-restriction phase remained elevated relative to the earlier pandemic stage. Future research using standardized, longitudinal designs is needed to accurately quantify the pandemic’s net effect on student mental health relative to pre-pandemic levels.

Nevertheless, our findings align with those of Li et al., who also used the IES-6, GAD-7, and PHQ-9 to assess acute stress, anxiety, and depression symptoms among Chinese students. They found that anxiety and depression rates increased, while acute stress declined, two months after the COVID-19 outbreak [2]. Previous studies have also reported that the prevalence of insomnia and SI continued to rise during the restriction period [11,30]. The trend in SI increase was consistent with findings by Liang, et al. [6], who also used the ninth item of the PHQ-9 to assess SI among Chinese college students during the pandemic. They reported that SI prevalence rose progressively from the outbreak period to the remission period and then into the normalized prevention and control phase. Our results are also in agreement with Peng et al. [8], who conducted a one-year longitudinal study and found an increase in anxiety and a decrease in fear during the restriction phase. Furthermore, our findings indicated that the prevalence of fear did not increase following the lifting of restrictions. This trend may reflect the public’s psychological adaptation to the pandemic, including the gradual development of psychological resilience, more rational understanding of epidemic information, and the psychological support offered by the government and broader society [8,31].

Notably, acute stress exhibited a unique V-shaped trend, distinct from other symptoms. At T1, acute stress levels were relatively high, likely due to the tremendous uncertainty introduced by the sudden outbreak, strict lockdown measures, and widespread health concerns [2,20]. At T2, although restrictions were still in place, the public had become more familiar with the virus, fear had decreased, and epidemic control procedures had become more routine. These changes may have contributed to a reduction in acute stress levels [8,32]. By T3, however, acute stress levels increased again, likely due to the widespread infection following policy relaxation. Students may have experienced direct and severe traumatic events, such as physical illness (e.g., fever, pain), witnessing classmates or friends becoming infected, or even enduring the serious illness or death of family and friends. The potential shortage of medical resources during this time may have also constituted a new and significant acute stressor. These findings are consistent with a previous study [15] which reported widespread PTSD symptoms among college students after the lifting of lockdown measures. In summary, trends in population-level prevalence across different time points suggest a sustained burden of these symptoms among college students over the study period, without clear evidence of recovery [2,6,30,33,34].

Demographic factors associated with mental symptoms in our study were consistent with previous literature. We found that educational background, sex, living environment, physical and mental health status, and history of psychotropic drug use were all associated with mental symptoms, which aligns with findings from earlier studies [9,20,32,35,36,37,38]. Furthermore, we identified several COVID-19-related risk factors. Being infected or experiencing post-infection sequelae were associated with increased risks of mental health problems. A recent study indicated that worsening distress and anxiety may result from COVID-19 symptoms such as fever, hypoxia, and cough, as well as from adverse treatment effects—such as insomnia caused by corticosteroids [39]. Our findings reveal a nuanced relationship between preventive behaviors and mental health. Compared with moderate prevention, much prevention was associated with higher risks of mental outcomes, while without prevention was linked to elevated anxiety, depression, and insomnia yet lower acute stress, fear, and suicidal ideation. This pattern suggests distinct psychological profiles: excessive prevention may reflect anxiety-driven hypervigilance and compulsive safety-seeking [40,41,42]. Conversely, without prevention may indicate avoidant coping, lower perceived threat, or emotional disengagement—factors that may buffer acute distress while coexisting with other vulnerabilities, particularly in individuals with pre-existing mental health conditions [41,43,44,45]. Together, these profiles underscore that preventive behaviors are not merely matters of compliance but are deeply intertwined with an individual’s psychological state and cognitive appraisal of the pandemic context. These highlight the importance of considering underlying anxiety, health literacy, and institutional trust in post-pandemic mental health support. In addition, the frequency of browsing COVID-19-related information online were linked to mental health symptoms, which is associated with increased distress, especially among individuals with high intolerance of uncertainty. For these individuals, more searching is linked to greater fear of COVID-19, which in turn predicts more engagement in preventive behaviors such as social distancing [42,46]. Misinformation and rumors circulating online during the pandemic have been shown to contribute to poor mental health outcomes [47]. We also found that students who were less concerned about their health after being infected with COVID-19 had a higher risk of experiencing depression, anxiety, insomnia, and SI. This phenomenon may be related to emotional numbing or avoidance behaviors, which are known to be associated with psychological symptoms [48,49]. Additionally, students who perceived greater negative impacts from the pandemic also displayed a higher risk of mental health issues. COVID-19 has led to household income shocks and disruptions in personal development, both of which negatively influence mental well-being [50,51]. Therefore, specific attention should be directed toward students who have not fully recovered from infection, those who reported substantial impacts, and those exhibiting maladaptive preventive or information-seeking behaviors during the pandemic. Given these findings, universities should implement targeted mental health interventions during public health emergencies. These should include routine mental health screenings for high-risk students (e.g., those experiencing persistent post-infection symptoms or displaying extreme preventive behaviors), and tailored counseling support for pandemic survivors. Furthermore, academic and financial assistance should be prioritized to help mitigate pandemic-related stressors.

Taken together, although most students in the current sample had been infected and restrictions had been relaxed compared to the earlier, more stringent lockdown phases, this relaxation was paradoxically accompanied by an increase in population-level mental health symptom prevalence. The observed patterns may reflect a complex interplay between adjustment processes to the post-restriction context and delayed or cumulative effects of the prolonged pandemic. On one hand, the elevation in symptoms may represent an adjustment disorder-like response to the abrupt societal and campus reopening, where students faced renewed academic pressures, reintegration into social and academic life, and exposure to widespread personal infection, all while processing the accumulated stressors of the preceding restrictive phases [52,53]. On the other hand, the sustained high prevalence of depression, anxiety, and suicidal ideation, may indicate delayed or cumulative psychological sequelae of prolonged pandemic-related stressors (e.g., chronic uncertainty, repeated disruptions, grief, and socioeconomic strain) [54,55]. The V-shaped pattern of acute stress—initially high, then decreasing during restrictions, and rising again after reopening—suggests that while some stress responses may adapt over time (as seen at T2), new acute stressors (e.g., personal illness, loss) can re-trigger significant distress, indicating that cumulative trauma exposure remains potent [56,57]. Thus, the phase of after restrictions likely introduced new adjustment demands while also acting as a catalyst that unveiled or exacerbated latent, cumulative distress from the preceding years. In conclusion, there remains a critical need for governmental and institutional authorities to reduce reinfection rates, restore socioeconomic stability, and ensure adequate access to both physical and mental healthcare for Chinese college students in the after-restriction era.

### 4.1. Limitations

Several limitations of the present study should be acknowledged. First, mental symptoms were assessed using self-reported questionnaires, which are not equivalent to clinical diagnoses. As such, no formal clinical assessments can be inferred from this study. In particular, suicidal ideation (SI) was assessed using a single item from the PHQ-9, which may limit the interpretability of the findings. However, it is important to acknowledge that this single-item, low-threshold approach likely increases the estimated prevalence of SI. Furthermore, this approach does not capture other important aspects of suicidality, such as suicidal planning or attempts. Consequently, the prevalence of SI reported in this study should be interpreted with caution; it represents a screening-positive rate for generalized risk rather than a clinical indicator of severe suicidality. Given the large-scale, anonymous online survey design, this method was deemed most appropriate to balance ethical considerations (minimizing potential distress from intrusive questioning while fulfilling the duty to identify risk) with research feasibility. For future studies aiming to assess the severity and multifaceted nature of suicidality more precisely, the use of comprehensive instruments such as the Suicidal Behaviors Questionnaire–Revised (SBQ-R) is recommended.

Second, and most pertinent to the study design, this was a serial cross-sectional study. Although we used standardized rates to enhance comparability across waves, the design does not permit causal inferences or the examination of within-individual trajectories. The observed changes in prevalence reflect population-level associations across different samples at different time points and should not be interpreted as evidence of individual-level worsening over time. Unmeasured cohort effects or sampling variability may also influence these trends. Longitudinal cohort studies are needed to elucidate causal relationships and individual trajectories.

Third, regarding sampling methodology, it is important to note that our reliance on online voluntary participation via QR codes and academic platforms may have introduced selection bias. This approach likely systematically excluded students with severe psychological distress (who may lack motivation or capacity to participate), those with limited internet access, and individuals disengaged from formal academic communication channels. As a result, the prevalence estimates reported here may be conservative and potentially underestimate the true burden of mental health symptoms in the broader college student population. This limitation also affects the generalizability of our findings, as the experiences of some of the most vulnerable student subgroups may not be adequately represented.

Fourth, the 22 participating universities in Guangdong Province were selected through a convenience sampling approach, which prioritized large-scale feasibility during a dynamic public health emergency. These institutions encompass a range of academic profiles, including comprehensive, science and engineering, medical, and normal universities, and are distributed across both the developed Pearl River Delta region and the less developed eastern, western, and northern areas of Guangdong. This mix partially captures the diversity of Chinese higher education institutions in terms of academic focus and student body composition. However, as a regional sample from one of China’s most populous and economically developed provinces, our findings may not be fully representative of the national landscape of college students. Factors such as institutional selectivity, regional economic disparities, and varying local pandemic control measures across China could influence mental health outcomes. Therefore, while our study provides critical insights into the psychological impact of evolving pandemic policies, caution is warranted when extrapolating the results to all college students in China.

It is important to emphasize that this study employed a serial cross-sectional design. Therefore, terms such as “worsening,” “persistence,” “long-term,” and “progression” used throughout the manuscript refer to changes in population-level prevalence estimates across independent samples at different phases of the pandemic. They do not imply within-individual deterioration or recovery over time, nor do they establish causal relationships. The findings reflect shifts in the overall burden of mental health symptoms at a group level in response to evolving pandemic contexts.

### 4.2. Implications for Practice and Policy

The persistent elevation of mental health symptoms among college students after restrictions were lifted underscores an urgent need for multilevel, actionable responses. Our findings point to several concrete steps for mental health services, campus programming, and public policy. At the university level, the high prevalence of symptoms—especially suicidal ideation—calls for enhanced, accessible support. Our identified risk groups (e.g., students with infection history, severe pandemic-related impacts on family income or personal development, and those with maladaptive preventive behaviors) offer a blueprint for targeted screening and outreach. Counseling centers should implement routine, proactive screenings that incorporate these factors, followed by stepped-care interventions: low-intensity supports (e.g., stress-management workshops, peer networks) for the general student body, and immediate, intensive care (e.g., individual therapy, crisis services) for high-risk individuals. Beyond clinical services, campuses must address the academic and socioeconomic stressors exacerbated by the pandemic. Interventions should include flexible academic accommodations, expanded financial aid and emergency grants, and career counseling tailored to a volatile job market. Equally important is fostering a mental health-positive campus culture through de-stigmatization campaigns and training staff to recognize and refer students in distress. These findings also carry broader public health policy implications. The sustained psychological burden necessitates a long-term, integrated strategy. Policymakers should fund mental health surveillance systems in educational settings, invest in training for university counseling staff, promote public messaging that normalizes help-seeking and builds resilience, and support cross-sectoral initiatives that tackle underlying social determinants such as economic insecurity and educational disruption.

In summary, translating our findings into action requires coordinated efforts across targeted campus strategies, strengthened clinical pathways, and supportive public policies. Prioritizing student mental well-being is both a clinical imperative and a vital investment in future societal resilience and productivity.

## 5. Conclusions

In summary, our findings show higher population-level prevalence rates after the lifting of restrictions of COVID-19 among Chinese college students in the prevalence of anxiety, depression, insomnia, and suicidal ideation. Acute stress exhibited a V-shaped trend, increasing after restrictions were lifted following a decrease during the restriction phase. Meanwhile, fear of COVID-19 has declined since the early stage of the pandemic. Despite the easing of restrictions, the current mental health status of Chinese college students remains a cause for concern. These results suggest that the pandemic phases were associated with changes in mental health symptom prevalence, underscoring the urgent need to prioritize prevention efforts and ensure access to mental health care.

## Figures and Tables

**Table 1:** Demographic information at T3 (15 March to 22 April 2023, after restrictions).

	No. (%)
Factor	(N = 130,285)
Grade	
undergraduates	123,030 (94.4)
postgraduates	7242 (5.6)
Sex	
male	53,564 (41.1)
female	76,720 (58.9)
living area	
urban	46,782 (35.9)
rural	83,503 (64.1)
severe somatic disease	
no	128,560 (98.7)
yes	1678 (1.3)
mental disorder	
no	127,581 (98.0)
yes	2657 (2.0)
psychotropic drugs	
no	125,116 (96.1)
yes	5169 (4.0)
COVID-19 infection	
uninfected	28,357 (21.8)
under infection	1084 (0.8)
infected	8467 (6.5)
recovery	92,377 (70.9)
Concerned more about self-health	
yes	46,413 (35.6)
no	83,872 (64.4)
Surfing information about COVID-19	
≤1 h	103,134 (79.2)
1–3 h	13,266 (10.2)
3–5 h	5714 (4.4)
≥5 h	8171 (6.3)
Impacts on family incomes	
severe impact	10,555 (8.1)
large impact	46,874 (36.0)
lesser impact	72,856 (55.9)
Impacts on academic or career development	
severe impact	10,999 (8.4)
large impact	47,249 (36.3)
lesser impact	72,037 (55.3)
COVID-19 prevention behaviors	
moderate prevention	15,856 (12.2)
much prevention	68,492 (52.6)
without prevention	45,937 (35.3)

Abbreviations: No., number.

**Table 2:** Crude and standardized prevalence rates of mental health outcomes at T3 (15 March to 22 April 2023, after restrictions).

	Participants, No. (%)							
	Characteristics		Mental Health Outcomes					
Characteristics	Target Population ^a^	Sample at T3	Anxiety (GAD-7 ≥ 10)	Depression (PHQ-8 ≥ 10)	Insomnia (YSIS ≥ 26)	Acute Stress (IES-6 ≥ 9)	Fear (CV-19FSS ≥ 4)	Suicidal Ideation (PHQ 9th > 0)
T3 Crude prevalence% (95% CI)	NA	130,272	13.8% (13.6–14.0)	20.4% (20.2–20.6)	11.8% (11.7–12.0)	19.8% (19.6–20.0)	16.9% (16.7–17.1)	32.3% (32.0–32.5)
Standard prevalence% (95% CI)	2,888,397	130,272	13.5% (13.3–13.7)	19.9% (19.6–20.1)	11.8% (11.6–12.0)	19.7% (19.5–20.0)	16.2% (16.0–16.4)	31.8% (31.5–32.1)
Undergraduates								
Male	1,273,609 (50.2)	50,359 (40.9)	6927 (6.8)	10,283 (9.0)	5527 (4.8)	12,854 (11.3)	8459 (7.4)	18,463 (16.2)
Female	1,266,170 (49.9)	72,671 (59.1)	10,349 (6.2)	15,325 (9.2)	8890 (5.4)	12,329 (7.4)	12,989 (7.8)	22,238 (13.4)
Postgraduates								
Male	170,890 (49.0)	3197 (44.2)	264 (0.5)	404 (0.8)	432 (0.8)	340 (0.6)	259 (0.5)	619 (1.2)
Female	177,728 (51.0)	4045 (55.9)	451 (0.7)	589 (0.9)	572 (0.9)	273 (0.4)	318 (0.5)	706 (1.1)

Note: ^a^ The college student population of Guangdong Province, 2020 to 2021, published by the Ministry of Education of the People’s Republic of China. Abbreviations: No., number; GAD-7, 7-item Generalized Anxiety Disorder Scale; PHQ-8, the 8-item Patient Health Questionnaire; YSIS, the 8-item Youth Self-Rating Insomnia Scale; IES-6, 6-item Impact of Event Scale; PHQ 9th, The 9th item of the 9-item Patient Health Questionnaire.

**Table 3:** Factors associated with poor mental health outcomes according to bivariate analysis.

		Anxiety		Depression		Insomnia		Acute Stress		Fear		Suicidal Ideation	
	No. (%)	(GAD-7 ≥ 10)		(PHQ-8 ≥ 10)		(YSIS ≥ 26)		(IES-6 ≥ 9)		(CV-19FSS ≥ 4)		(PHQ 9th > 0)	
Factor	(N = 130,285)	No.	*p*	No.	*p*	No.	*p*	No.	*p*	No.	*p*	No.	*p*
Grade													
undergraduates	123,030 (94.4)	17,276	***	25,608	***	14,417	***	25,183	***	21,448	***	40,701	***
postgraduates	7242 (5.6)	715		993		1004		613		577		1325	
Sex													
male	53,564 (41.1)	7193	***	10,691	***	5960	***	13,196	***	8721	***	19,085	***
female	76,720 (58.9)	10,800		15,913		9462		12,603		13,307		22,944	
living area													
urban	46,782 (35.9)	6656	**	10,090	***	5541	0.952	11,161	***	9361	***	16,394	***
rural	83,503 (64.1)	11,338		16,515		9881		14,638		12,668		25,636	
Severe somatic disease													
no	128,560 (98.7)	17,569	***	26,043	***	15,096	***	25,453	0.941	21,417	***	41,261	***
yes	1678 (1.3)	408		540		312		331		587		733	
Mental disorder													
no	127,581 (98.0)	16,997	***	25,316	***	14,576	***	25,367	***	21,187	***	40,558	***
yes	2657 (2.0)	979		1263		837		419		815		1437	
Psychotropic drugs													
no	125,116 (96.1)	16,242	***	24,325	***	13,962	***	24,751	0.384	20,460	***	39,244	***
yes	5169 (4.0)	1752		2280		1460		1048		1569		2786	
COVID-19 infection													
uninfected	28,357 (21.8)	3941	***	5877	***	3087	***	6271	***	5521	***	9699	***
under infection	1084 (0.8)	332		409		139		358		439		695	
infected	8467 (6.5)	2020		2819		1760		2333		2258		3777	
recovery	92,377 (70.9)	11,701		17,500		10,436		16,837		13,811		27,859	
Concerned more about self-health													
yes	46,413 (35.6)	7709	***	11,492	***	6366	***	7977	***	5877	***	17,122	***
no	83,872 (64.4)	10,285		15,113		9056		17,822		16,152		24,908	
Surfing information about COVID-19													
≤1 h	103,134 (79.2)	13,319	***	19,875	***	12,174	***	16,636	***	14,961	***	29,910	***
1–3 h	13,266 (10.2)	2146		3036		1314		4809		3729		5862	
3–5 h	5714 (4.4)	944		1371		671		1938		1439		2611	
≥5 h	8171 (6.3)	1585		2323		1263		2416		1900		3647	
Impacts on family incomes													
severe impact	10,555 (8.1)	2534	***	3571	***	1913	***	3344	***	3219	***	5035	***
large impact	46,874 (36.0)	7395		11,151		6091		11,149		9454		17,109	
lesser impact	72,856 (55.9)	8065		11,883		7418		11,306		9356		19,886	
Impacts on academic or career development													
Severe impact	10,999 (8.4)	2924	***	3859	***	2113	***	3501	***	3228	***	5271	***
large impact	47,249 (36.3)	7779		11,525		6212		11,247		9713		17,511	
lesser impact	72,037 (55.3)	7291		11,221		7097		11,051		9088		19,248	
COVID-19 prevention behaviors													
moderate prevention	15,856 (12.2)	2731	***	3659	***	1786	***	5164	***	5437	***	6678	***
much prevention	68,492 (52.6)	8783		13,141		7622		14,468		12,217		21,824	
without prevention	45,937 (35.3)	6480		9805		6014		6167		4375		13,528	

Note: ** *p* < 0.01, *** *p* < 0.001; GAD-7. Abbreviations: No., number; 7-item Generalized Anxiety Disorder Scale; PHQ-8, the 8-item Patient Health Questionnaire; YSIS, the 8-item Youth Self-Rating Insomnia Scale; IES-6, 6-item Impact of Event Scale; PHQ 9th, The 9th item of the 9-item Patient Health Questionnaire.

**Table 4:** Factors associated with poor mental health outcomes according to multivariate logistic regression.

Factor	Anxiety	Depression	Insomnia	Acute Stress	Fear	Suicidal Ideation
	(GAD-7 ≥ 10)	(PHQ-8 ≥ 10)	(YSIS ≥ 26)	(IES-6 ≥ 9)	(CV-19FSS ≥ 4)	(PHQ 9th > 0)
	Adjusted OR (95%CI)	Adjusted OR (95%CI)	Adjusted OR (95%CI)	Adjusted OR (95%CI)	Adjusted OR (95%CI)	Adjusted OR (95%CI)
Grade						
undergraduates	1 (Reference)					
postgraduates	0.75 (0.69, 0.81) ***	0.67 (0.62, 0.72) ***	1.29 (1.20, 1.38) ***	0.43 (0.39, 0.46) ***	0.49 (0.45, 0.54) ***	0.50 (0.47, 0.53) ***
Sex						
male	1 (Reference)					
female	1.16 (1.12, 1.20) ***	1.15 (1.11, 1.18) ***	1.19 (1.14, 1.23) ***	0.59 (0.57, 0.61) ***	1.13 (1.09, 1.16) ***	0.80 (0.78, 0.82) ***
Living area						
urban	1 (Reference)					
rural	0.97 (0.94, 1.00)	1.02 (0.99, 1.05)	0.98 (0.95, 1.02)	1.25 (1.21, 1.29) ***	1.19 (1.15, 1.23) ***	1.09 (1.06, 1.11) ***
Severe somatic disease						
no	1 (Reference)					
yes	1.12 (0.99, 1.27)	1.10 (0.98, 1.24)	1.09 (0.95, 1.24)	0.74 (0.65, 0.84) ***	1.77 (1.58, 1.99) ***	1.03 (0.93, 1.15)
Mental disorder						
no	1 (Reference)					
yes	1.67 (1.50, 1.85) ***	1.71 (1.54, 1.88) ***	1.67 (1.50, 1.86) ***	0.72 (0.63, 0.81) ***	1.30 (1.16, 1.46) ***	1.36 (1.23, 1.50) ***
Psychotropic drugs						
no	1 (Reference)					
yes	2.42 (2.24, 2.61) ***	2.35 (2.19, 2.52) ***	2.27 (2.09, 2.46) ***	1.09 (1.00, 1.19) *	1.80 (1.66, 1.95) ***	2.10 (1.96, 2.26) ***
COVID-19 infection						
uninfected	1 (Reference)					
under infection	2.08 (1.81, 2.39) ***	1.81 (1.59, 2.07) ***	1.02 (0.84, 1.22)	1.27 (1.11, 1.46) ***	2.11 (1.84, 2.41) ***	2.59 (2.27, 2.96) ***
infected	1.83 (1.72, 1.95) ***	1.83 (1.73, 1.93) ***	2.01 (1.88, 2.14) ***	1.42 (1.34, 1.50) ***	1.49 (1.40, 1.58) ***	1.55 (1.48, 1.64) ***
recovery	0.95 (0.92, 0.99) *	0.94 (0.91, 0.98) ***	1.06 (1.02, 1.11) **	0.89 (0.86, 0.93) ***	0.83 (0.80, 0.87) ***	0.91 (0.88, 0.94) ***
Concerned more about self-health						
yes	1 (Reference)					
no	1.48 (1.43, 1.53) ***	1.56 (1.51, 1.60) ***	1.28 (1.24, 1.33) ***	0.90 (0.87, 0.93) ***	0.72 (0.70, 0.74) ***	1.54 (1.50, 1.58) ***
Surfing information about COVID-19						
≤1 h	1 (Reference)					
1–3 h	1.28 (1.22, 1.35) ***	1.24 (1.18, 1.29) ***	0.86 (0.81, 0.91) ***	2.40 (2.30, 2.50) ***	1.75 (1.68, 1.83) ***	1.79 (1.73, 1.87) ***
3–5 h	1.31 (1.22, 1.41) ***	1.31 (1.22, 1.39) ***	1.01 (0.93, 1.10)	2.42 (2.28, 2.57) ***	1.69 (1.58, 1.80) ***	1.98 (1.87, 2.09) ***
≥5 h	1.47 (1.38, 1.56) ***	1.51 (1.44, 1.59) ***	1.29 (1.21, 1.38) ***	1.97 (1.87, 2.08) ***	1.55 (1.46, 1.64) ***	1.78 (1.70, 1.87) ***
Impacts on family incomes						
lesser impact	1 (Reference)					
severe impact	1.56 (1.47, 1.65) ***	1.75 (1.66, 1.84) ***	1.48 (1.39, 1.58) ***	1.57 (1.48, 1.65) ***	1.79 (1.69, 1.89) ***	1.62 (1.54, 1.70) ***
large impact	1.28 (1.23, 1.33) ***	1.38 (1.34, 1.43) ***	1.23 (1.19, 1.28) ***	1.34 (1.30, 1.39) ***	1.35 (1.31, 1.40) ***	1.30 (1.26, 1.33) ***
Impacts on academic or career development						
lesser impact	1 (Reference)					
severe impact	2.53 (2.39, 2.68) ***	2.23 (2.12, 2.35) ***	1.85 (1.74, 1.97) ***	1.72 (1.63, 1.81) ***	1.80 (1.70, 1.90) ***	1.79 (1.71, 1.88) ***
large impact	1.62 (1.56, 1.68) ***	1.58 (1.53, 1.63) ***	1.33 (1.28, 1.38) ***	1.39 (1.35, 1.44) ***	1.44 (1.39, 1.49) ***	1.41 (1.38, 1.45) ***
COVID-19 prevention behaviors						
moderate prevention	1 (Reference)					
much prevention	1.17 (1.11, 1.22) ***	1.05 (1.01, 1.10) *	0.90 (0.85, 0.96) ***	1.43 (1.38, 1.49) ***	1.95 (1.87, 2.03) ***	1.29 (1.24, 1.34) ***
without prevention	1.13 (1.09, 1.17) ***	1.15 (1.11, 1.19) ***	1.18 (1.14, 1.23) ***	0.62 (0.60, 0.64) ***	0.56 (0.54, 0.58) ***	0.88 (0.86, 0.90) ***

Note: * *p* < 0.05, ** *p* < 0.01, *** *p* < 0.001. Multicollinearity among predictors was assessed using variance inflation factors (VIFs) prior to model fitting. All VIF values were below 5, indicating no substantial multicollinearity that would compromise the model estimates. Abbreviations: OR, Odds Ratio; GAD-7, 7-item Generalized Anxiety Disorder Scale; PHQ-8, the 8-item Patient Health Questionnaire; YSIS, the 8-item Youth Self-Rating Insomnia Scale; IES-6, 6-item Impact of Event Scale; PHQ 9th, The 9th item of the 9-item Patient Health Questionnaire.

## Data Availability

The data presented in this study are available on request from the corresponding author due to confidentiality.

## Supplementary Materials

The following supporting information can be downloaded at: https://www.mdpi.com/article/10.3390/healthcare14030339/s1, Table S1: Crude and standardized Prevalence rates of mental health outcomes at T1 (from 3 to 10 February, 2020, onset of COVID-19); Table S2: Crude and standardized Prevalence rates of mental health outcomes at T2 (10 to 18 June, 2021, during restrictions).
