# Supplementary material for: Mental Health Symptoms Among Chinese College Students Following the Lifting of COVID-19 Restrictions: A Serial Cross-Sectional Study in Guangdong Province, China
Source: Healthcare (Basel). 2026 Jan 29;14(3):339. doi: 10.3390/healthcare14030339 (PMC12896657; doi:10.3390/healthcare14030339)
Supplement: Supplementary file 1 [file healthcare-14-00339-s001.zip › healthcare-4073373-supplementary.pdf]

Table S1. Crude and standardized Prevalence rates of mental health outcomes at T1 (from 3 to 10 February, 2020, onset of COVID-19).

	Participants, No. (%)							
	Characteristics		Metal health outcomes					
Characteristic	Target population [a]	Study sample	Anxiety (GAD-7≥10)	Depression (PHQ-8≥10)	Insomnia (YSIS≥26)	Acute stress (IES-6≥9)	Fear (Panic-12≥4)	Suicidal ideation (PHQ 9th>0)
Crude prevalence %(95%CI)	NA	164101	2.6% (2.6-2.7)	6.4% (6.3-6.5)	2.8% (2.7-2.9)	35.1% (34.9-35.4)	49.4% (49.2-49.7)	8.5% (8.3-8.6)
Standard prevalence of Guangdong %(95%CI)	2888397	164101	2.6% (2.5-2.7)	6.0% (5.9-6.2)	2.7% (2.6-2.8)	35.4% (35.1-35.8)	47.4% (47.1-47.8)	8.3% (8.1-8.4)
Undergraduates								
Male	1273609 (50.2)	58199 (37.0)	1358 (1.0)	3046 (2.3)	1515 (1.2)	20619 (15.6)	24245 (18.4)	5178 (3.9)
Female	1266170 (49.9)	99270 (63.0)	2774 (1.2)	7035 (3.1)	2903 (1.2)	34478 (15.2)	53836 (23.8)	8392 (3.7)
Postgraduates								
Male	170890 (49.0)	2257 (34.0)	55 (0.1)	87 (0.2)	47 (0.1)	817 (2.1)	840 (2.2)	129 (0.3)
Female	177728 (51.0)	4375 (66.0)	160 (0.2)	281 (0.4)	100 (0.1)	1736 (2.4)	2185 (3.1)	210 (0.3)

Note: [a]The college student population of Guangdong Province, 2020 to 2021, published by the Ministry of Education of the People's Republic of China.

Abbreviations: GAD-7, 7-item Generalized Anxiety Disorder Scale; PHQ-8, the 8-item Patient Health Questionnaire; YSIS, the 8-item Youth Self-Rating Insomnia Scale; IES-6, 6-item Impact of Event Scale; PHQ 9th, The 9th item of the 9-item Patient Health Questionnaire.

Table S2. Crude and standardized Prevalence rates of mental health outcomes at T2 (10 to 18 June, 2021, during restrictions).

	Participants, No. (%)							
	Characteristics		Metal health outcomes					
Characteristic	Target population [a]	Study sample	Anxiety (GAD-7≥10)	Depression (PHQ-8≥10)	Insomnia (YSIS≥26)	Acute stress (IES-6≥9)	Fear (Panic-12≥4)	Suicidal ideation (PHQ 9th>0)
Crude prevalence %(95%CI)	NA	86767	3.8% (3.6-3.9)	8.2% (8.0-8.4)	7.7% (7.5-7.9)	18.9% (18.6-19.1)	16.4% (16.1-16.6)	16.5% (16.3-16.8)
Standard prevalence of Guangdong %(95%CI)	2888397	86767	3.8% (3.6-4.1)	8.1% (7.8-8.3)	7.5% (7.3-7.8)	18.7% (18.3-19.1)	16.2% (15.6-16.6)	16.2% (15.9-16.6)
Undergraduates								
Male	1273609 (50.2)	34194 (39.9)	1232 (1.6)	2657 (3.4)	2232 (2.9)	7545 (9.7)	5090 (6.6)	6406 (8.3)
Female	1266170 (49.9)	51530 (60.1)	1981 (1.7)	4351 (3.7)	4373 (3.7)	8690 (7.4)	9021 (7.7)	7827 (6.7)
Postgraduates								
Male	170890 (49.0)	427 (40.9)	16 (0.2)	31 (0.4)	32 (0.4)	57 (0.8)	50 (0.7)	48 (0.7)
Female	177728 (51.0)	616 (59.1)	34 (0.3)	49 (0.5)	47 (0.5)	77 (0.8)	25 (0.3)	61 (0.6)

Note: [a]The college student population of Guangdong Province, 2020 to 2021, published by the Ministry of Education of the People's Republic of China.

Abbreviations: GAD-7, 7-item Generalized Anxiety Disorder Scale; PHQ-8, the 8-item Patient Health Questionnaire; YSIS, the 8-item Youth Self-Rating Insomnia Scale; IES-6, 6-item Impact of Event Scale; PHQ 9th, The 9th item of the 9-item Patient Health Questionnaire.
